# Probable hepatic capillariosis and hydatidosis in an adolescent from the late Roman period buried in Amiens (France)

**DOI:** 10.1051/parasite/2014010

**Published:** 2014-02-28

**Authors:** Gholamreza Mowlavi, Sacha Kacki, Jean Dupouy-Camet, Iraj Mobedi, Mahsasadat Makki, Majid Fasihi Harandi, Saied Reza Naddaf

**Affiliations:** 1 Department of Medical Parasitology and Mycology, School of Public Health, Tehran University of Medical Sciences PO Box 6446 Tehran 14155 Iran; 2 PACEA, UMR 5199, Anthropologie des Populations Passées et Présentes, Université de Bordeaux Bâtiment B8, Allée Geoffroy St Hilaire, CS 50023 33615 Pessac Cedex France; 3 Service de Parasitologie-Mycologie, Hôpital Cochin Assistance Publique Hôpitaux de Paris, Université Paris Descartes 27 Faubourg St Jacques 75014 Paris France; 4 Research Center for Hydatid Disease in Iran, Kerman University of Medical Sciences Kerman 76169-14111 Iran; 5 Department of Parasitology, Pasteur Institute of Iran 69 Pasteur Avenue Tehran 13169-43551 Iran; 6 Center for Research of Endemic Parasites of Iran (CREPI), Tehran University of Medical Sciences PO Box 6446 Tehran 14155 Iran

**Keywords:** Paleoparasitology, Capillariosis, Hydatidosis, *Calodium*, *Capillaria*, *Echinococcus granulosus*

## Abstract

Two calcified objects recovered from a 3rd to 4th-century grave of an adolescent in Amiens (Northern France) were identified as probable hydatid cysts. By using thin-section petrographic techniques, probable *Calodium hepaticum* (syn. *Capillaria hepatica*) eggs were identified in the wall of the cysts. Human hepatic capillariosis has not been reported from archaeological material so far, but could be expected given the poor level of environmental hygiene prevalent in this period. Identification of tissue-dwelling parasites such as *C. hepaticum* in archaeological remains is particularly dependent on preservation conditions and taphonomic changes and should be interpreted with caution due to morphological similarities with *Trichuris* sp. eggs.

## Abstract (fr)

Deux objets calcifiés découverts dans la tombe d’un adolescent du 3^ème^-4^ème^ siècle à Amiens (France) ont été identifiés comme des kystes hydatiques probables. En utilisant des techniques pétrographiques, des œufs de *Calodium hepaticum* (syn. *Capillaria hepatica*) ont été identifiés dans la paroi de ces kystes. La capillariose hépatique n’a jamais été rapportée de restes archéologiques mais n’est pas inattendue compte tenu du bas niveau d’hygiène de cette époque. L’identification de parasites tissulaires comme *C. hepaticum* dans des vestiges archéologiques est particulièrement dépendante des conditions de conservation et de changements taphonomiques et doit être interprétée avec circonspection en raison des similitudes morphologiques de ces œufs avec ceux du trichocéphale (*Trichuris* sp.).

## Introduction

Paleoparasitology is the identification of parasites found in archaeological material. Sometimes parasitic eggs are relatively well preserved in latrines or mummified bodies, but their identification can be difficult. We report and discuss here the probable occurrence of human hepatic capillariosis associated with hydatidosis in an adolescent from the late Roman period buried in Amiens (Northern France). Although hydatidosis, particularly of the liver, still remains a problem of public health worldwide [20], human capillariosis is quite infrequent, but could have been more prevalent in ancient times due to greater contact with rodents and low hygiene in general. *Calodium hepaticum* (syn. *Capillaria hepatica*, Bancroft, 1893), the causative agent of hepatic capillariosis, is a parasitic nematode of rodents and other mammalian species, including humans. The adult worms invade the liver parenchyma of the host and lay numerous eggs in the surrounding tissues [21]. Hepatic capillariosis is a rare infection in humans and is associated with septal fibrosis of the liver, which leads to cirrhosis in severe infections [16, 22]. Infection transmission mostly occurs after ingestion of embryonated eggs, which are disseminated in the soil following decomposition of parasitized dead animals, or in the environment following defecation by predators or scavengers [28]. Diagnosis requires a liver biopsy or post-mortem examination to demonstrate the presence of eggs [27]. The recovery of capillariid eggs from archaeological remains has been recorded: e.g., in human coprolites from the Neolithic inhabitants of Chalain (Jura, France) [7], in a pellet belonging to a Patagonian bird of prey from 6500 years ago [18], in Argentine rodent coprolites from 8000 years ago [33], in non-human organic remains from the French Medieval site of Charavines [5], and from the archaeological layers of “Place d’Armes” (Namur, Belgium) in which seven historical strata exist from the Roman period up to modern times [13].

## Materials and methods

**Figure 1.**
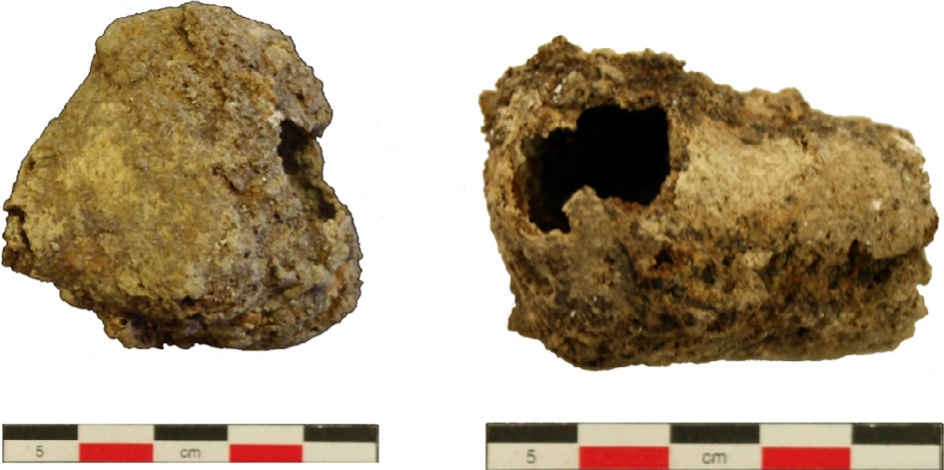
Cysts obtained from the grave of a late Roman adolescent in Amiens, France.

During an excavation in 2009 in the city of Amiens (Northern France) investigators recovered two ovoid cyst-like objects from the grave of an adolescent of unknown sex (Fig. 1). The burial was part of a small necropolis dating back to the Late Roman period (3rd–4th century AD). Although the skeleton was poorly preserved, all bones were connected and the cystic objects were obtained from the thoraco-abdominal region. The calcified objects, measuring 46 × 46 × 37 mm and 47 × 33 × 32 mm, respectively, exhibited a rough and irregular outer surface of brown/black color. Several holes in the outer wall revealed a hollow interior, with some powder inside. Contrary to the external surface, the internal surface was smooth. During the initial anthropological study, the aspect of the cysts indicated likely liver hydatidosis. In order to confirm this hypothesis, the cysts and the containing elements were analyzed by X-ray diffraction (XRD) and element analysis by X-ray fluorescence (XRF). X-ray analyses were performed at the Faculty of Sciences, Tarbiat Modares University, Tehran, Iran. To examine the material for parasites, the powder inside the cysts was rehydrated using trisodium phosphate (TSP) aqueous solution, as recommended [8]. In addition, tissue sections were prepared from the wall of the cysts by means of thin-section petrography, a well-established method in geology [10, 31, 37], but with some modifications. Four pieces of the specimen measuring about 1–2 × 1.5 cm were precisely sawed from the cysts, using a 50-cm-long steel wire. Due to the fragility of the specimen, samples were impregnated with resin (Epofix) in a vacuum chamber for 3 h and embedding was continued for another 8 h in the same chamber. Impregnated blocks were smoothed manually on one side and mounted on glass slides with Epofix resin. Grinding to a thickness of 1–2 mm and 50-60 μm was performed by a diamond blade (Microplan-TR) and diamond grinding wheel (Microplan-TR), respectively. Finally, to achieve the 30-μm thickness required for light transmission, silicon carbide powder (Mesh Nos. 320, 600, 800, and 1000) was used manually. At the end of each grinding step, coarse material was removed with tap water. Section slide mounting was performed using mixed low-viscosity resin and a cover slip under 0.5 kg pressure for 2 h. In addition, some parts of the cysts were thoroughly pulverized and rehydrated in TSP solution for 10 days. The parasite eggs were microscopically detected, photomicrographed, and measured using a calibrated microscope.

## Results

**Figure 2.**
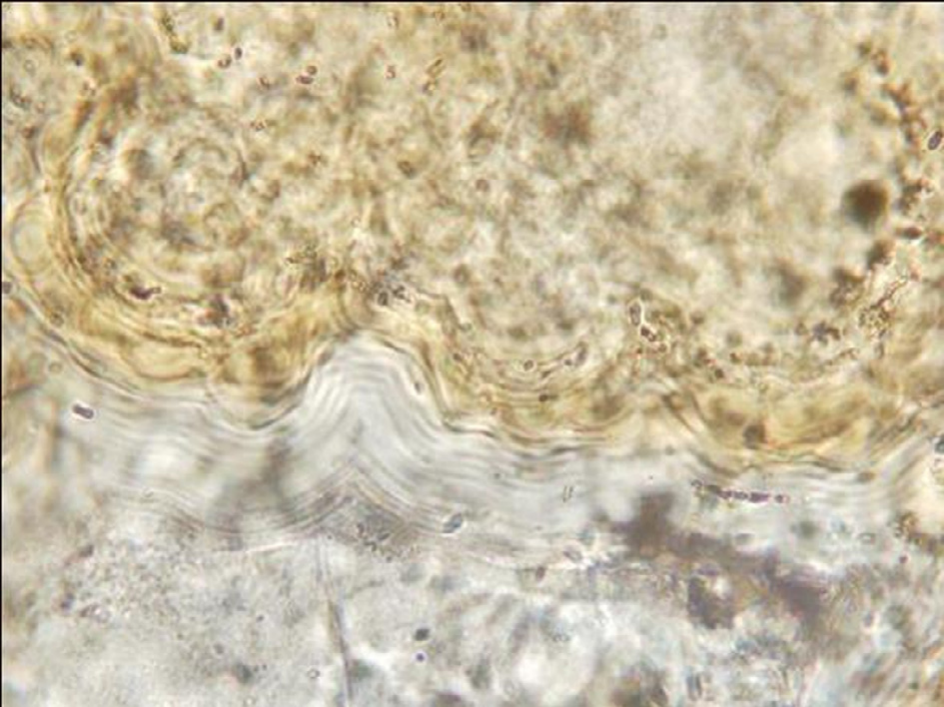
Laminated layers observed on a thin section of the cyst wall suggesting hydatidosis.

**Figure 3.**
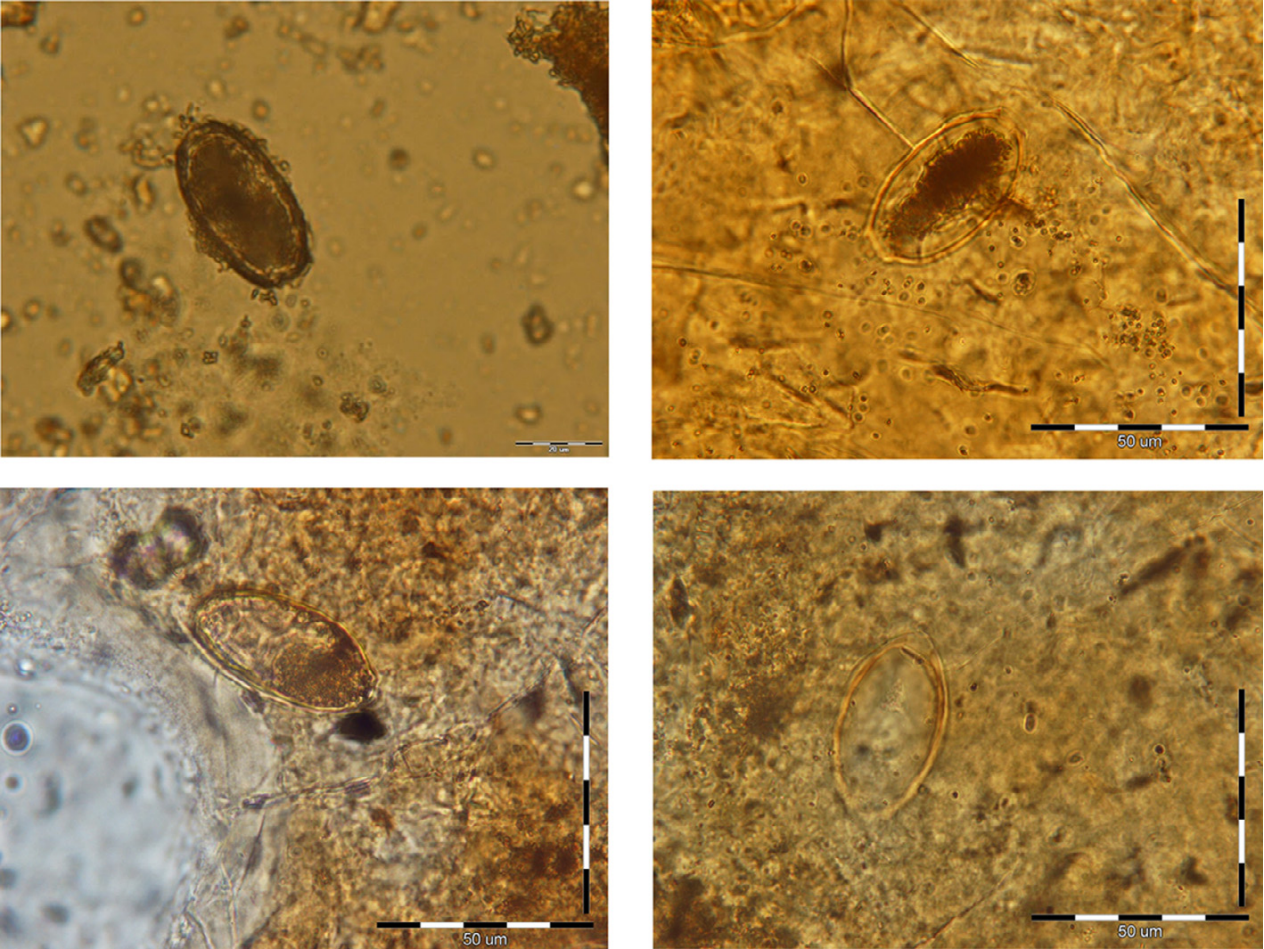
Probable *Calodium hepaticum* eggs detected in the rehydrated pulverized cyst (a) and in thin-section slides (b–d). Scale bars = 50 μm, a–d: from top to base.

For the first cyst, XRD clearly detected the presence of both calcite and apatite (data not shown). These components were also present in the second cyst, together with other crystallographic species (quartz and iron). The XRF analyses showed that the main chemical elements of both cysts were calcium oxide and phosphorus oxide (Table 1). The high concentration of phosphorus in both samples is highly consistent with an organic origin. In addition, in both cysts, the stoichiometry P/Ca was compatible with that of hydroxyapatite, which can be considered as the main component of the cyst (or even the only one, if the few other elements are due to telluric pollution). Precise detection of larval rostellar hooks, which could have characterized hydatid cysts, was negative, but in one section a faded structure resembling a hydatid cyst laminated layer was observed (Fig. 2). In addition, capillariid eggs were detected in all 15 thin-section slides and in the rehydrated pulverized cyst. Eggs in the section slides (Figs. 3b–3d) were more transparent than those observed in the rehydrated pulverized cyst (Fig. 3a). The mean length and width of the eggs were 46.7 and 25 μm, respectively, and these eggs were tentatively identified as *C. hepaticum*. No parasite remains or eggs were seen in the powder from inside the cysts.

**Table 1.** Chemical analysis by X-ray fluorescence of samples from the two cysts.

%	Na_2_O	MgO	AlO_3_	SiO_2_	P_2_O_5_	SO_3_	CaO	Fe_2_O_3_	Sr
Cyst 1	0.19	0.42	0.15	0.85	37.9	0.3	59.6	0.39	0.07
Cyst 2	0.24	0.58	2.1	20.4	18.9	0.36	54.2	1.7	0.08

## Discussion

These findings support the biological origin of the two cysts, as apatite is the main component of bones and teeth [38] and is also commonly found in tissue calcifications linked to various pathological conditions [25]. The mineral composition of the cysts, their location, their morphology, and the observation of a laminated layer are compatible with the diagnosis of liver hydatidosis [3, 25]. However, in the absence of larval rostellar hooks, only DNA-based techniques would allow a definitive diagnosis. Hydatid cysts frequently undergo calcification, and numerous ancient cases have been reported from Europe [3, 24, 30, 39]. The morphological characteristics of these published examples – ovoid, hollow concretions with a smooth internal surface and an irregular external surface – are quite similar to those of the two calcified objects reported here. These morphological features rule out most of the other diagnostic options, such as calcified objects that are solid (e.g., urinary or renal calculi, uterine leiomyoma, dermoid cysts), that are not expected to be so large (e.g., calcified lymph nodes), or which exhibit imprints of vascularization (e.g., calcified ovarian cysts) [2, 23]. Calcification of internal organs, induced by a normal taphonomic process after a long burial, seems quite unlikely according to the experience of one of the authors of the present work. Finally, although we cannot definitely exclude that the cysts underwent migration within the body during its decomposition, their location in the thoraco-abdominal region of the skeleton is again compatible with liver hydatidosis. The attachment of the cysts to the liver parenchyma would explain why *C. hepaticum* eggs were found in the wall of these cysts. A review of the literature shows that the only archaeological materials in which capillariid eggs have been detected so far are human coprolites, sediment of sewage structures, and garbage dumps [4, 9, 13]. Within these types of archaeological remains a definite diagnosis of human hepatic capillariosis could not be concluded. The finding of capillariid eggs in human coprolites can be explained by the transitional passing of parasite eggs, following the ingestion of an infected animal liver [17], as reported among Indians of the Amazonian region [12]. The proof of hepatic capillariosis in archaeological remains would only be possible through the examination of remaining tissues in mummified bodies, or by analyzing exceptional samples like the ones isolated in Amiens. In all 15 slides, several *C. hepaticum* eggs were detected possessing the main characters of *Calodium hepaticum* eggs, such as a double barrel-shape, pitted shell, polar plugs not extending beyond the outline shell and radiation between the external layers [19, 21, 34]. However, due to ultra-structural changes that may occur to helminth eggs over a long period of time [6], all morphological and morphometric features were not simultaneously observed in individual eggs. The mean length and width of the recovered eggs (46.7 and 25 μm) are in agreement with the findings reported by others [21, 26]. The thoraco-abdominal location of the examined cysts, besides the findings presented here, are altogether in favor of a true hepatic capillariosis. Actually, *Capillaria*-like eggs have already been found in human graves, but in that report the authors suggested secondary contamination by feces of rodents [15]. In our case, this type of contamination can be excluded, as the eggs were found in the wall of the cysts. Another possibility could be the contamination of the cysts by *Trichuris trichiura* (Linnaeus, 1771) eggs from the intestine of the young adolescent. However, *Trichuris* sp. eggs have thicker shells and are usually very well preserved (in particular the polar plugs) as, for example, in the famous Ötzi mummy dating from the Neolithic times [1], or in the French Medieval site of Charavines [5]. The possibility of infection with *Eucoleus aerophilus* (Creplin, 1839), another capillariid parasite with morphologically similar eggs, could be ruled out as eggs of this parasite are larger (60–83 × 25–40 μm) and have asymmetric polar plugs [36]. Human hepatic capillariosis can be acquired under various circumstances, including high exposure to rodent populations and poor hygiene [35]. Soil, vegetables, and water contaminated by rat feces containing embryonated eggs are known sources of infection [16, 29]. In ancient times due to the lack of public health awareness the incidence of hepatic capillariosis could have been more significant than today, as only 37 case reports of human *C. hepaticum* infection have been reported up to 2010 [17, 26]. Moreover, new findings of *C. hepaticum* infection in mice in human-inhabited houses in Portugal [32] and the respective prevalence of 36% and 44% *Capillaria* infection of rats in Italy [11] and Marseille [14], respectively, support the idea of the possible frequent occurrence of human hepatic capillariosis in the 3rd–4th century AD. This work revealed that the petrographic thin-section technique is an efficient tool for identification of hepatic capillariosis and other tissue-dwelling parasites in very old organic remains, as well as in fossilized specimens. This current observation illustrates quite well the difficulties of differential diagnosis in paleoparasitology, related to the resemblance of the eggs of different species of capillariids. It underlines the usefulness of a precise registration of the position of samples within the skeleton and the possible influence of taphonomic processes in the modification of egg morphology. Finally, this case of probable liver hydatidosis is certainly the most ancient reported so far in France.
